# Fatal Pneumonia Associated With a Novel Genotype of Human Coronavirus OC43

**DOI:** 10.3389/fmicb.2021.795449

**Published:** 2022-01-14

**Authors:** Susanna Kar Pui Lau, Kenneth Sze Ming Li, Xin Li, Ka-Yan Tsang, Siddharth Sridhar, Patrick Chiu Yat Woo

**Affiliations:** Department of Microbiology, Li Ka Shing Faculty of Medicine, The University of Hong Kong, Hong Kong, Hong Kong SAR, China

**Keywords:** human coronavirus OC43, fatal, pneumonia, novel genotype, hypervirulence

## Abstract

Since its first discovery in 1967, human coronavirus OC43 (HCoV-OC43) has been associated with mild self-limiting upper respiratory infections worldwide. Fatal primary pneumonia due to HCoV-OC43 is not frequently described. This study describes a case of fatal primary pneumonia associated with HCoV-OC43 in a 75-year-old patient with good past health. The viral loads of the respiratory tract specimens (bronchoalveolar lavage and endotracheal aspirate) from diagnosis to death were persistently high (3.49 × 10^6^–1.10 × 10^10^ copies/ml). HCoV-OC43 at a 6.46 × 10^3^ copies/ml level was also detected from his pleural fluid 2 days before his death. Complete genome sequencing and phylogenetic analysis showed that the present HCoV-OC43 forms a distinct cluster with three other HCoV-OC43 from United States, with a bootstrap value of 100% and sharing 99.9% nucleotide identities. Pairwise genetic distance between this cluster and other HCoV-OC43 genotypes ranged from 0.27 ± 0.02% to 1.25 ± 0.01%. In contrast, the lowest pairwise genetic distance between existing HCoV-OC43 genotypes was 0.26 ± 0.02%, suggesting that this cluster constitutes a novel HCoV-OC43 genotype, which we named genotype I. Unlike genotypes D, E, F, G, and H, no recombination event was observed for this novel genotype. Structural modeling revealed that the loop with the S1/S2 cleavage site was four amino acids longer than other HCoV-OC43, making it more exposed and accessible to protease, which may have resulted in its possible hypervirulence.

## Introduction

Coronaviruses (CoVs) are classified into four genera, *Alphacoronavirus*, *Betacoronavirus*, *Gammacoronavirus*, and *Deltacoronavirus*. AlphaCoVs and betaCoVs are found exclusively in mammals, whereas gammaCoVs and deltaCoVs mainly infect birds (Woo et al., 2012; Lau et al., 2021). Among all the CoVs, seven are known to infect humans. Three of them (all betaCoVs), including Severe Acute Respiratory Syndrome CoV (SARS-CoV) and SARS-CoV-2 that emerged from China in 2002/2003 and 2019, respectively, and Middle East Respiratory Syndrome CoV (MERS-CoV) that emerged from the Middle East in 2012, probably originated from recent animal-to-human transmission and resulted in highly fatal pneumonia (Lau et al., 2005; Zaki et al., 2012; Lau et al., 2020). The other four, namely human CoV (HCoV)-OC43 (a betaCoV), HCoV-229E (an alphaCoV), HCoV-NL63 (an alphaCoV), and HCoV-HKU1 (a betaCoV), are HCoVs that are primarily associated with upper respiratory infections (Hamre and Procknow, 1966; McIntosh et al., 1967; van der Hoek et al., 2004; Woo et al., 2005a).

Since its first discovery in 1967, HCoV-OC43 has been reported to be associated with mild self-limiting upper respiratory infections worldwide (McIntosh et al., 1967). Fatal primary pneumonia due to HCoV-OC43 is not frequently described. In 2011, based on phylogenetic analysis of the complete RNA-dependent RNA polymerase (RdRp), spike (S), and nucleocapsid (N) genes, we subclassified HCoV-OC43 into genotypes A, B, C and D, and showed that genotype D was generated through natural recombination (Lau et al., 2011). Four additional genotypes, namely E, F, G, and H, were described (Zhang et al., 2015; Oong et al., 2017; Zhu et al., 2018) in the last 10 years. This article describes a patient with fatal pneumonia associated with HCoV-OC43. Complete genome sequencing and phylogenetic analysis revealed that it is distinct from all these eight genotypes of HCoV-OC43. Based on these results, we propose a novel genotype of HCoV-OC43, named genotype I. The possible pathogenic mechanism for this virus leading to fatal infection is also discussed.

## Materials and Methods

### Patient and Clinical Specimens

Various clinical samples, including bronchoalveolar lavage, tracheal aspirate, sputum, endotracheal aspirate, and pleural fluid, were collected at different time points from the patient. The respiratory samples were then detected for respiratory pathogens, including 17 viruses and 4 bacteria, by BioFire^®^ FilmArray^®^ Respiratory Panel 2 (RP2) and as well as bacterial/fungal culture and PCR. Due to the recent emergence of COVID-19, the samples were also tested for SARS-CoV-2 retrospectively. The collection and use of clinical samples and data were approved by the Institutional Review Board of the University of Hong Kong/Hospital Authority Hong Kong West Cluster (UW 16-365 20-07-2016).

### RNA Extraction

Viral RNA was extracted from the clinical specimens of the patient using QIAamp Viral RNA Mini Kit (QIAgen, Hilden, Germany). The RNA was eluted in 60 μl of Buffer AVE and was used as the template for RT-PCR.

### Complete Genome Sequencing

Complete genome sequencing of the HCoV-OC43 from the patient (HK19-01) was performed using primers and strategies as we described previously (Lau et al., 2011). The viral RNA was reverse transcribed to cDNA by a combined random priming and oligo(dT) priming strategy. The cDNA was amplified by degenerate primers designed by multiple alignments of available HCoV-OC43 complete genome sequences. Additional primers were designed from the results of the first and subsequent rounds of sequencing. These primer sequences are available on request. The 5′ ends of the viral genomes were confirmed by rapid amplification of cDNA ends using the 5′/3′ RACE kit (Roche, Germany). Sequences were assembled and manually edited to produce the final sequence of the viral genome.

### Quantitative Real-Time RT-PCR

Viral loads of HCoV-OC43 in different clinical specimens collected from the patient were performed by quantitative real-time RT-PCR targeting the N gene. RNA was amplified in a LightCycler instrument with SuperScript III Platinum One-Step Quantitative RT-PCR System (Invitrogen, San Diego, CA, United States) using forward primer 5′-CGAT GAGGCTATTCCGACTAGGT-3′, reverse primer 5′-CCTTCC TGAGCCTTCAATATAGTAACC-3′ and probe 5′-(FAM)-TCC GCCTGGCACGGTACTCCCT-(BHQ-1)-3′, with the following cycling protocol: 30 min at 50°C for reverse transcription, followed by 2 min at 95°C and 50 cycles of 15 s at 95°C and 30 s at 55°C. For quantitative analysis, a reference standard was prepared using the pCRII-TOPO vector (Invitrogen, San Diego, CA, United States) containing the target sequence. A calibration curve was generated by serial 10-fold dilutions equivalent to 2.21 × 10^2^–2.21 × 10^9^ copies per reaction mixture parallel with test samples.

### Phylogenetic, Recombination, and Genome Analysis

Opening reading frames (ORFs) of the HCoV-OC43 genome encoding proteins were predicted using ORFfinder (NIH, United States) and compared to available complete HCoV-OC43 genomes. Phylogenetic analysis of the complete HCoV-OC43 genomes was performed using the maximum likelihood method using MEGA X (Kumar et al., 2018), with the best-fit model (TN93 + G) selected and bootstrap values calculated from 1,000 trees. Pairwise genetic distances between HCoV-OC43 genotypes based on the complete genome sequences were calculated using MEGA X.

Recombination analysis was performed using Simplot version 3.5.1 as described previously (Lole et al., 1999). Bootscan analysis for a recombination event was performed on a gapless nucleotide alignment of the HCoV-OC43 genome sequences of different genotypes generated by MEGA X with the proposed novel genotype I as the query sequence. A sliding window of 1,000 nucleotides and a step size of 200 nucleotides were used as the scanning settings.

### Estimation of Synonymous and Non-synonymous Substitution Rates

The number of synonymous substitutions per synonymous site, Ks, and the number of non-synonymous substitutions per non-synonymous site, Ka, for each coding region was calculated using the Nei-Gojobori method (Jukes-Cantor) in MEGA X.

### Protein Structural Modeling

The structure of HCoV-OC43 S glycoprotein trimer was predicted using a web-based homology-modeling server, SWISS-MODEL (Waterhouse et al., 2018). BLASTp search was performed against Protein Data Bank (PDB) with the default parameters to find suitable templates for homology modeling. Based on the higher sequence identity, QMEAN Z-score, coverage, and lower e-value, the crystal structure of the HCoV-OC43 S (PDB code: 6NZK) was selected as the template.

### Nucleotide Sequence Accession Number

The genome sequence of the HCoV-OC43 from the patient was deposited in GenBank sequence database under accession no. MW938760.

## Results

### Patient

**FIGURE 1:**
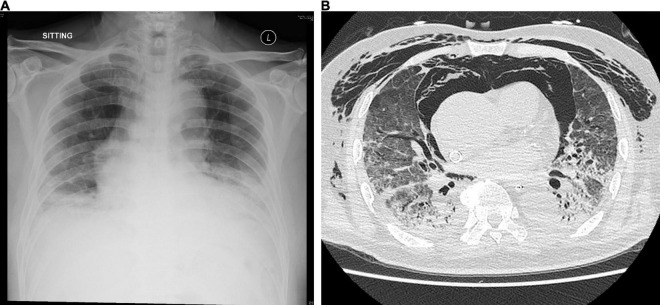
**(A)** Chest radiograph on admission showing bilateral lower zone reticulonodular shadows and **(B)** CT scan of the thorax on day 15 showing mixed consolidative and atelectatic changes in the dependent regions of both lungs.

A 75-year-old Chinese man with good past health was admitted to the hospital in August 2019 because of 1-week breath shortness. He also complained of dry cough, decreased appetite, and subjective low-grade fever. On admission, the temperature was 37.2°C. The blood pressure was 128/70 mmHg and pulse rate 91/min. Chest examination showed bilateral lower zone fine crepitation. The chest radiograph revealed bilateral lower zone reticulonodular shadows (Figure 1A). The total white cell count was 8.9 × 10^9^/L (reference range 3.7–9.3 × 10^9^/L), with mild neutrophilia of 7.7 × 10^9^/L (reference range 1.8–6.2 × 10^9^/L) and lymphopenia of 0.6 × 10^9^/L (reference range 1.0–3.2 × 10^9^/L). There was mild hyponatremia with a serum sodium level of 135 mmol/L (range 136–145 mmol/L). The renal function test was normal. The serum bilirubin was normal, but both alkaline phosphatase and alanine transaminase were high at 212 U/L (reference range 30–120 U/L) and 87 U/L (reference range < 50 U/L), respectively. A clinical and radiological diagnosis of community acquired pneumonia was made. A nasopharyngeal swab was obtained for multiplex RT-PCR detection of influenza virus A, B and C, adenovirus, parainfluenza virus 1–4, respiratory syncytial virus, human metapneumovirus and enterovirus/rhinovirus. Sputum was collected for bacterial culture and urine for *Streptococcus pneumoniae* and *Legionella* antigen detection. Empirical intravenous ceftriaxone was commenced. All the preliminary microbiological investigations showed negative results.

Despite the treatment, his conditions gradually deteriorated, with increased oxygen requirement and serial chest radiographs showed increased bilateral reticular shadows. On day 7, ceftriaxone was stopped, and intravenous piperacillin-tazobactam and oral doxycycline were started. Intravenous hydrocortisone was also started. However, his condition continued to deteriorate. He required high-flow oxygen on day 11 and was intubated on day 12. He also developed hypotension requiring inotrope support. Due to persistently low oxygenation despite maximum ventilatory support, he was put on venous-venous extracorporeal membrane oxygenation (VV-ECMO) and transferred to our teaching hospital on day 13 of admission.

Bronchoalveolar lavage was performed on day 14. Rapid respiratory pathogen detection using the BioFire^®^ FilmArray^®^ Respiratory Panel 2 (RP2) was positive for HCoV-OC43 only and was negative for other viruses. RT-PCR for SARS-CoV-2 was also negative. The HCoV-OC43 from the patient in this study was designated as HK19-01. Bacterial culture showed scanty growth of *Pseudomonas aeruginosa*. Fungal culture, mycobacterial culture, and PCR detection of *Mycobacterium tuberculosis*, *Legionella pneumophila*, *Pneumocystis jirovecii*, cytomegalovirus, herpes simplex virus and varicella zoster virus were all negative. A computed tomography scan of the thorax showed mixed consolidative/atelectatic changes in the dependent regions of both lungs (Figure 1B). The antibiotics were switched to intravenous meropenem, levofloxacin and vancomycin. However, the patient remained VV-ECMO-dependent and developed oliguric renal failure requiring continuous veno-venous hemofiltration. He developed *Candida albicans* fungemia on day 22, and intravenous anidulafungin was added. Despite all the treatment, his clinical condition continued to deteriorate with multiorgan failure. He finally succumbed on day 30.

### Viral Load

The viral load of the respiratory tract samples (bronchoalveolar lavage and endotracheal aspirate) from diagnosis to his death were persistently high (3.49 × 10^6^–1.10 × 10^10^ copies/ml) (Table 1). HCoV-OC43 at a level of 6.46 × 10^3^ copies/ml was also detected from his pleural fluid 2 days before his death.

**TABLE 1:** Viral loads of different respiratory samples collected from the patient infected with the novel HCoV-OC43 genotype at different time points.

Collection date	Specimen	Viral load (copy no./ml)
Day 14	Bronchoalveolar lavage	3.13 × 10^9^
Day 18	Endotracheal aspirate	1.10 × 10^10^
Day 19	Endotracheal aspirate	8.21 × 10^8^
Day 22	Endotracheal aspirate	8.38 × 10^8^
Day 28	Pleural fluid	6.46 × 10^3^
Day 29	Bronchoalveolar lavage	3.49 × 10^6^

### Phylogenetic Analysis

**FIGURE 2:**
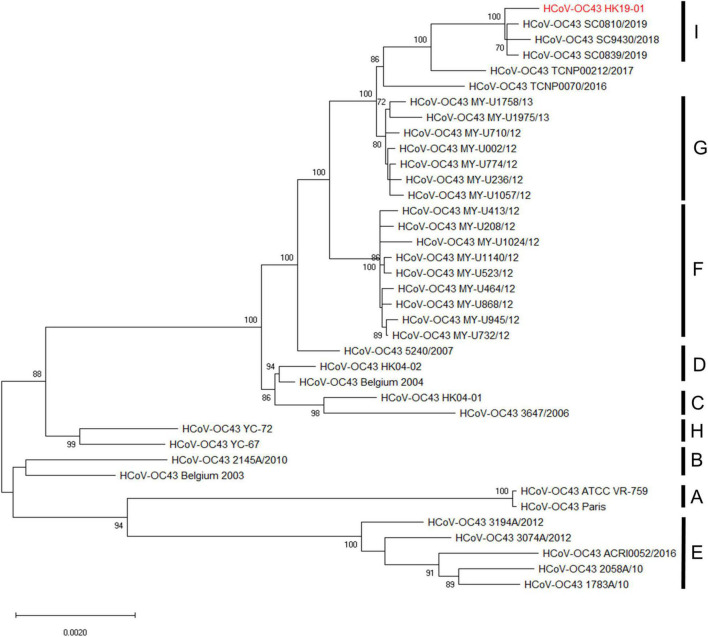
Phylogenetic analysis of HCoV-OC43 whole-genome sequences. The tree was constructed by the maximum-likelihood method with the best-fit TN93 + G model, and bootstrap values were calculated from 1,000 trees. A total of 30,726 nucleotide positions were used for the analysis and bootstrap values over 70% were shown in the nodes. The scale bar indicates the estimated number of substitutions per 500 nucleotides. The genome sequence of HCoV-OC43 HK19-01 from the patient was indicated by red color.

Phylogenetic analysis using the complete genome sequences showed that HCoV-OC43 HK19-01 forms a distinct cluster with three other HCoV-OC43 from United States (GenBank accession numbers MN306041, MN306042, and MN306053), with a bootstrap value of 100% and sharing 99.9% nucleotide identities (Figure 2).

### Genome Analysis

**FIGURE 3:**
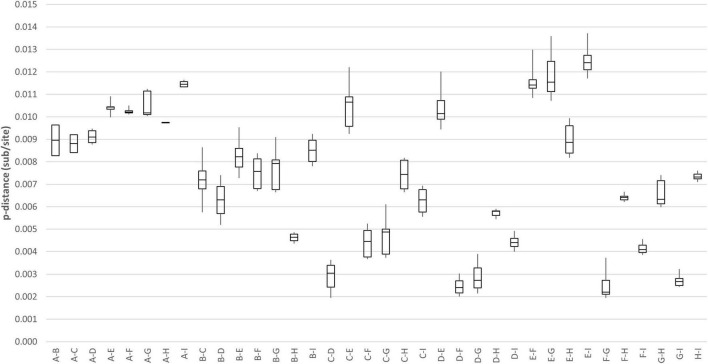
Estimation of pairwise genetic distances between genotype I and other existing genotypes of HCoV-OC43 based on the whole-genome sequences.

Genome analysis revealed a distinct four-amino-acid (768ASDI771) insertion in the S2 region just downstream to the S1/S2 cleavage site of HCoV-OC43 HK19-01 (Table 2). Another four-amino-acid (167KLKN170) insertion in the hemagglutinin-esterase protein present in some HCoV-OC43 of genotype G is also present (Table 2). The three other HCoV-OC43 from United States in this cluster shared nearly the same genome sequence as that of HCoV-OC43 HK19-01 except without certain amino acid substitution/insertion in nsp2, nsp3, nsp12, S, and ns5a proteins as in HCoV-OC43 HK19-01 (Table 2). Previously, a new genotype was proposed when the pairwise genetic distance of the whole genome sequence between a certain strain and the other genotypes was higher than that among the existing genotypes (Oong et al., 2017; Zhu et al., 2018). In this study, pairwise genetic distance between this cluster and other HCoV-OC43 genotypes ranged from 0.27 ± 0.02% to 1.25 ± 0.01%. In contrast, the lowest pairwise genetic distance between existing HCoV-OC43 genotypes was 0.26 ± 0.02% (Figure 3), suggesting that this cluster constitutes a novel HCoV-OC43 genotype, which we named genotype I. Bootscan analysis did not reveal any recombination leading to this new genotype I (Supplementary Figure 1).

**TABLE 2:** Signature amino acid substitutions of the novel HCoV-OC43 genotype.

Genotype	Strain	ORF1a					ORF1b		HE					S							ns5a
		nsp2		nsp3			nsp12	nsp14													
						1				Insertion between 166 and 167						Insertion between 762 and 763					
		*1	5	1	4	7								2	5					8	
		5	4	4	2	9	7	4						6	0					8	9
		0	3	3	4	7	1	4	5					2	4					7	9
A	ATCC VR-759	S	A	Q	P	A	V	G	P	–	–	–	–	L	P	–	–	–	–	S	T
B	Belgium 2003	.	.	.	.	.	.	.	.	–	–	–	–	.	.	–	–	–	–	.	.
C	HK04-01	.	.	.	.	.	.	.	.	–	–	–	–	.	.	–	–	–	–	.	.
D	HK04-02	.	.	.	.	.	.	.	.	–	–	–	–	.	.	–	–	–	–	.	.
E	3194A/2012	.	.	.	.	.	.	.	.	–	–	–	–	.	.	–	–	–	–	.	.
F	MY-U1024/12	.	.	.	.	.	.	.	.	–	–	–	–	.	.	–	–	–	–	.	.
G	MY-U1057/12	.	.	.	.	.	.	.	.	–	–	–	–	.	.	–	–	–	–	.	.
	MY-U774/12	.	.	.	.	.	.	.	.	–	–	–	–	.	.	–	–	–	–	.	.
	TCNP0070/2016	.	.	.	.	.	.	.	.	K	L	K	N	.	.	–	–	–	–	.	.
	TCNP00212/2017	.	.	.	.	.	.	.	.	K	L	K	N	.	.	–	–	–	–	.	.
H	YC-72	.	.	.	.	.	.	.	.	–	–	–	–	.	.	–	–	–	–	.	.
I	SC9430/2018	L	.	R	.	S	.	S	S	K	L	K	N	P	S	–	–	–	–	A	.
	SC0839/2019	L	.	R	.	S	.	S	S	K	L	K	N	P	S	–	–	–	–	A	.
	SC0810/2019	L	.	R	.	S	.	S	S	K	L	K	N	P	S	–	–	–	–	A	.
	HK19-01	L	V	R	H	S	I	S	S	K	L	K	N	P	S	A	S	D	I	A	N

*Amino acid position of respective viral proteins of different HCoV-OC43 genotypes.

### Estimation of Synonymous and Non-synonymous Substitution Rates

Using all four available proposed genotype I HCoV-OC43 genome sequences for analysis, the Ka/Ks ratios for the various coding regions were calculated (Table 3). The highest Ka/Ks ratios in this genotype were observed at S (1.0000), suggesting that the S gene in genotype I is under higher selection pressure. The Ka/Ks ratio for the S gene in genotype I is much higher than that in other genotypes (0.0426–0.4731), in which the Ka/Ks ratio for the S gene dropped to 0.2827 when using 54 strains of different genotypes for analysis.

**TABLE 3:** Estimation of non-synonymous and synonymous substitution rates of the novel HCoV-OC43 genotype.

Gene	K_a_	K_s_	K_a_/K_s_
nsp1	0	0.0060	0
nsp2	0.0011	0	
nsp3	0.0003	0.0028	0.1071
nsp4	0	0.0028	0
nsp5	0	0.0024	0
nsp6	0	0	
nsp7	0	0	
nsp8	0	0	
nsp9	0	0	
nsp10	0	0	
nsp11	0	0	
nsp12	0.0002	0.0008	0.2500
nsp13	0	0.0012	0
nsp14	0	0.0029	0
nsp15	0.0006	0	
nsp16	0	0.0050	0
ns2a	0	0.0029	0
HE	0.0010	0.0034	0.2941
S	0.0011	0.0011	1.0000
ns5a	0.0020	0	
E	0.0026	0	
M	0	0	
N	0	0.0016	0

## Discussion

We describe a patient with fatal pneumonia caused by a novel genotype of HCoV-OC43. Although HCoV-OC43 is much more commonly associated with upper respiratory tract infections, its role as the causative agent of pneumonia in the present case is evident by the persistent detection of high viral loads in multiple lower respiratory tract samples throughout the course of the patient’s illness, even before the death of the patient. This is in contrast to cases of respiratory viral infections complicated by fatal secondary pneumonia, in which the viral loads often decrease gradually. Still, pneumonia caused by pyogenic bacteria, such as *Streptococcus pneumoniae* and *Staphylococcus aureus*, is the cause of severe sepsis, respiratory failure, and death of the patients. Moreover, no significant bacterial pathogen causing secondary bacterial pneumonia was isolated from the present patient, and he failed to respond to broad-spectrum antibiotic therapy. The HCoV-OC43 HK19-01 from the patient represents a novel genotype because the pairwise genetic distance between this novel genotype and other genotypes of HCoV-OC43 is higher than the lowest pairwise genetic distance between all the known HCoV-OC43 genotypes. However, unlike genotypes D, E, F, G and H, no recombination event was observed for this novel genotype. It is of note that although occasional fatalities as a result of primary viral pneumonia have been described in human betaCoVs such as HCoV-OC43 or HCoV-HKU1 (Vabret et al., 2003; Woo et al., 2005b), hypervirulence has not been reported in other genotypes of HCoV-OC43 or the three genotypes of HCoV-HKU1 (Woo et al., 2006; Lau et al., 2011; Zhang et al., 2015; Oong et al., 2017; Zhu et al., 2018). Nevertheless, this could be a result of our lack of knowledge on the molecular epidemiology of HCoVs, as genotyping of HCoVs is generally not performed in routine clinical microbiology laboratories.

**FIGURE 4:**
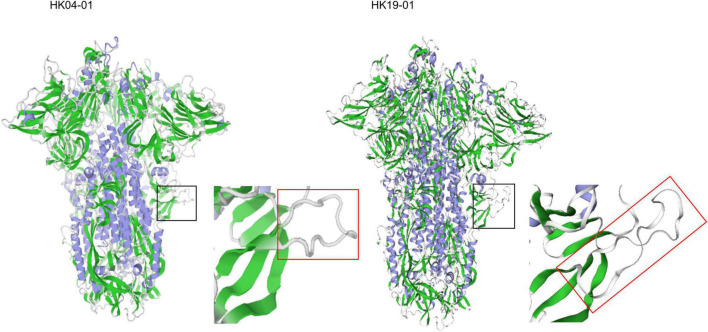
Structure of the HCoV-OC43 spike glycoprotein trimer predicted by SWISS-MODEL homology modeling. Models were generated using the structure of HCoV-OC43 (Protein Data Bank code 6NZK). The black square zone is magnified, and the red rectangle shows loop containing the S1/S2 furin protease cleavage site (aa residues 756–772). The left and right panel represents HCoV-OC43 HK04-01 and HK19-01, respectively.

The possible hypervirulence of this strain or novel genotype of HCoV-OC43 may be related to the four-amino-acid insertion in the S2 region just downstream to the S1/S2 cleavage site. It has been well-recognized that modifying the amino acid residues at the S1/S2 junction or improving the conditions that facilitate cleavage of the S proteins in SARS-CoV-2 and MERS-like-CoV, respectively, enhances their infectivities (Andersen et al., 2020; Menachery et al., 2020; Johnson et al., 2021). This in turn generates a higher viral load and may make the viruses more pathogenic. As for HCoV-OC43 HK19-01, although it has already possessed the optimal amino acid residues at the cleavage site, an 768ASDI771 insertion was present just downstream to the S1/S2 junction. Three-dimensional structural modeling showed that the loop that contained the S1/S2 cleavage site is 17 amino acids long in HCoV-OC43 HK19-01, as compared to 13 amino acids in other HCoV-OC43 (Figure 4). We speculate that this could have made the S1/S2 cleavage site more exposed and accessible to the protease. Notably, the corresponding loop with the S1/S2 cleavage site in SARS-CoV-2 (15 amino acids) is also four aa longer than other SARSr-CoVs (11 amino acids) (Lemmin et al., 2020). Further experiments will reveal the relative importance of the length of the loop in comparison to the amino acid composition of the cleavage site itself and other factors. On the contrary, the disease information and patient condition of the other three viruses from United States of this novel genotype are lacking. Hence, it cannot be excluded that the current fatal case may be exceptional.

## Data Availability Statement

The datasets presented in the study are deposited in the GenBank sequence database, accession number MW938760.

## Ethics Statement

The studies involving human participants were reviewed and approved by the Institutional Review Board of the University of Hong Kong/Hospital Authority Hong Kong West Cluster (UW 16-365 20-07-2016). The patients/participants provided their written informed consent to participate in this study. Written informed consent was obtained from the individual(s) for the publication of any potentially identifiable images or data included in this article.

## Author Contributions

SL, KL, XL, and PW conceived and designed the experiments and drafted the manuscript. KL, XL, and K-YT performed the experiments. SL, KL, XL, K-YT, SS, and PW contributed to the analysis. All authors provided critical feedback and revised the manuscript.

## Conflict of Interest

The authors declare that the research was conducted in the absence of any commercial or financial relationships that could be construed as a potential conflict of interest.

## Publisher’s Note

All claims expressed in this article are solely those of the authors and do not necessarily represent those of their affiliated organizations, or those of the publisher, the editors and the reviewers. Any product that may be evaluated in this article, or claim that may be made by its manufacturer, is not guaranteed or endorsed by the publisher.
