# Supplementary material for: Fatal Pneumonia Associated With a Novel Genotype of Human Coronavirus OC43
Source: Front Microbiol. 2022 Jan 14;12:795449. doi: 10.3389/fmicb.2021.795449 (PMC8795699; doi:10.3389/fmicb.2021.795449)
Supplement: Supplementary file 1 [file Data_Sheet_1.PDF]

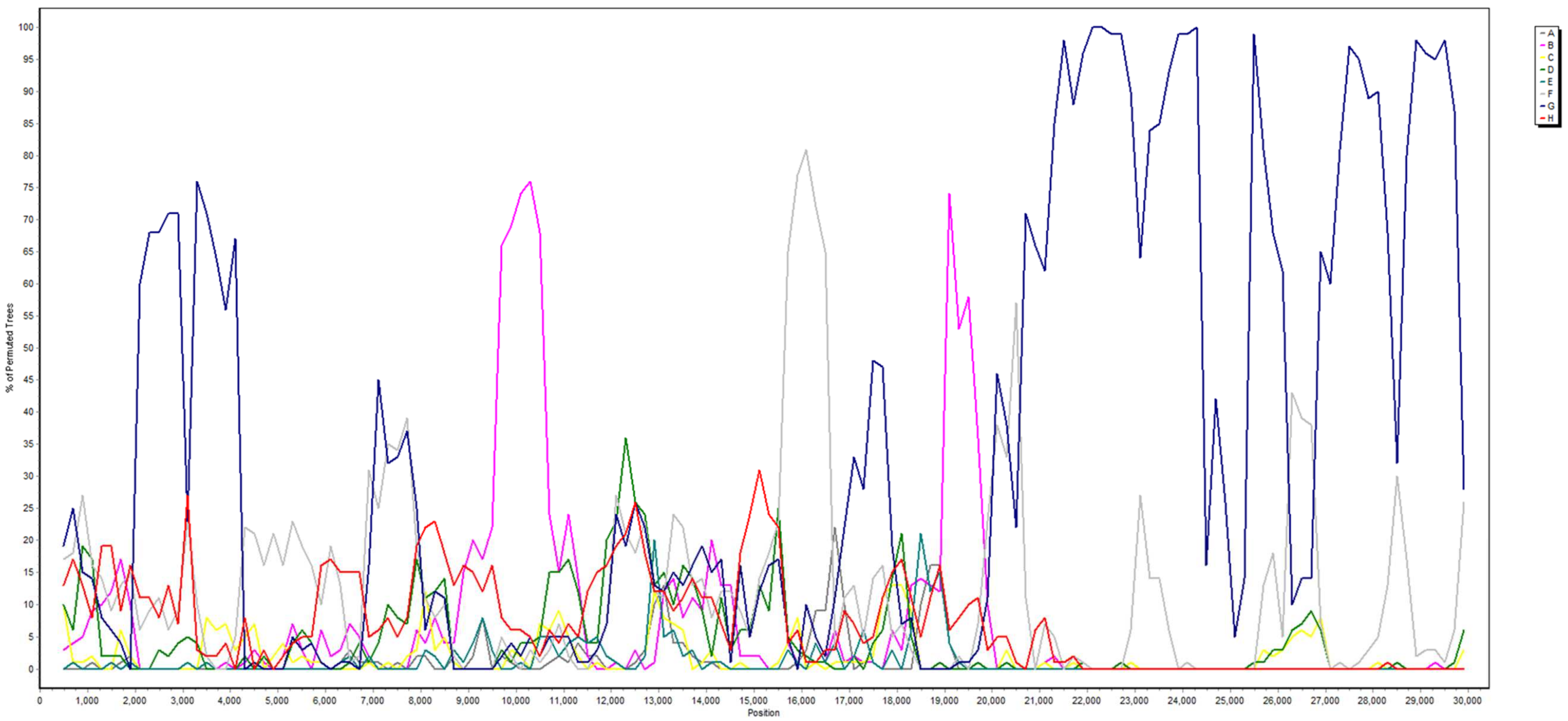

Supplementary Figure 1 Bootscan analysis using the genome sequence of HCoV-OC43 HK19-01 (proposed genotype I) as the query sequence. Bootscanning was conducted with Simplot version 3.5.1 (F84 model; window size, 1,000 bp; step, 200 bp) on a gapless nucleotide alignment.
